# A Comparison of Miller Straight Blade and Macintosh Blade Laryngoscopes for Intubation in Morbidly Obese Patients

**DOI:** 10.3390/jcm13030681

**Published:** 2024-01-24

**Authors:** Pawel Ratajczyk, Przemysław Kluj, Bartosz Szmyd, Julia Resch, Piotr Hogendorf, Adam Durczynski, Tomasz Gaszynski

**Affiliations:** 1 Department of Anaesthesiology and Intensive Therapy, Medical University of Lodz, 90-419 Lodz, Poland; przemyslaw.kluj@umed.lodz.pl (P.K.); julia.resch@stud.umed.lodz.pl (J.R.); tomasz.gaszynski@umed.lodz.pl (T.G.); 2 Department of Neurosurgery and Neuro-Oncology, Medical University of Lodz, 90-419 Lodz, Poland; bartosz.szmyd@umed.lodz.pl; 3 Department of General and Transplant Surgery, Medical University of Lodz, 90-419 Lodz, Poland; piotr.hogendorf@umed.lodz.pl (P.H.); adam.durczynski@umed.lodz.pl (A.D.)

**Keywords:** Miller laryngoscope, Macintosh laryngoscope, glottis view, Cormac–Lehane scale, POGO scale

## Abstract

The primary objective of this study was to demonstrate whether the Miller blade laryngoscope could provide better visualization of the vocal cords in morbidly obese patients than the Macintosh blade laryngoscope. The secondary objective was to identify the patient-measured factors associated with better visualization of the vocal cords when using the Miller vs. Macintosh blade, as well as whether the application of external pressure might improve the visibility of the glottis during intubation. A prospective, observational study encompassing 110 patients with a BMI > 40 undergoing elective bariatric surgery and intubation procedure was performed. The evaluation of the vocal cords was performed according to the Cormack–Lehane scale and POGO scale in the same patient during intubation, performed with a Miller and a Macintosh blade laryngoscope, in a random matter. The following parameters were assessed: body weight, height, BMI, neck circumference, thyromental distance, sternomental distance, mouth opening, and Mallampati scale and their impact on visualization of the vocal cords using the Miller blade without the application of external pressure. The Miller blade provides an improved view of the glottis compared to the Macintosh blade measured with both the Cormac–Lehane scale (45 (40.91%) without external pressure application on the larynx, and 18 (16.36%) with external pressure application on the larynx) and the POGO scale (45 (40.91%) without external pressure application on the larynx, and 19 (17.27%) with external pressure application on the larynx). The application of laryngeal pressure improved the view of the glottis. Among the measured features, a significant improvement in the visibility of the glottis could be found in patients with a BMI over 44.244 kg/m^2^ and a neck circumference over 46 cm. To conclude, the usage of the Miller blade improves the visibility of the glottis compared to the Macintosh blade in morbidly obese patients. The recommendation to use the Miller blade in this group of patients requires further investigation, taking into account the effectiveness of the intubation. Trial Registration: NCT05494463.

## 1. Introduction

Over the last few years, the number of obese patients has been constantly increasing worldwide. According to WHO (World Health Organization) data from 2008, over 10% of adults were obese, with a BMI of >30 kg/m^2^, and in 2030 the predicted number may even increase to 38%. The number of people with a BMI > 30 varies over the following countries: in the USA 33%, in Canada 24%, in Great Britain 23%, and in Japan and Korea, 4% each [1]. Obesity is a challenge for anesthesiologists as it may often be associated with difficult mask ventilation and potential difficult intubation [2]. In such patients, reduced compliance of the chest wall, together with increased risk of aspiration of gastric contents into the lung, can be expected [3]. Due to the reduced functional residual capacity (FRC), this group of patients is very sensitive to hypoxia, especially in periods without ventilation. Data from Great Britain show that obese patients account for 42% of patients with airway obstruction complications leading to administration to the intensive care unit, brain damage, and even death. All of these factors contribute to the fact that airway management in this group of patients is associated with a high risk of complications, and demands especially high intubation skills [4,5]. The use of an appropriate device to open the airway can significantly shorten the period of apnea and therefore reduce the percentage of possible complications [6,7]. Currently, the most common device used for endotracheal intubation is the Macintosh laryngoscope and the related intubation technique consisting in lifting the epiglottis to visualize the opening to the airways [8,9]. In a situation where dealing with a patient with a large tongue and a relatively small amount of space in the oral cavity or pharynx, e.g., in obese patients with a flaccid, aplastic, or sunken epiglottis, the attempt to lift it using a classical Macintosh laryngoscope or one of its various variants may not lead to significant results [10]. The usage of video laryngoscopes, which are often used as a rescue device during difficult intubation, may also be unsatisfactory in this group of patients, regardless of how much anesthesiologists may modify its angle [11,12,13,14,15]. Applying external pressure to the larynx may also not improve intubation conditions. In this situation, the intubation technique used for the Miller laryngoscope, which consists in placing the tip of the blade under the laryngeal surface of the epiglottis, or even the use of this laryngoscope can be useful. This approach allows for good visualization of the glottis and reduces the number of blind intubations or the usage of guiding devices, as described by Ueda and Arai [11].

Many articles have published reports that the use of a Miller blade enables better visualization of the glottis than the use of the Macintosh blade [16]. Authors suggest that less tissue needs to be displaced to obtain an adequate view of the glottis when using the Miller blade. Characteristically in obese patients, the supraglottic area shows significant anatomical variations. The larynx often appears to be located in front of the “line of sight” and is also called the “anterior” larynx [16]. These anatomical features are also found in pediatric patients where the Miller blade is already used as the gold standard [17,18]. However, there are no data on the success of its use in morbidly obese patients with a BMI > 40.

Our study investigates the hypothesis that the Miller blade may provide a better visualization of the glottis than the Macintosh blade in morbidly obese patients. The visualization of the glottis was assessed based on the Cormack–Lehane scale (CL scale) as well as the newer, and many authors have found, better scale, POGO scale (PG scale) [19]. It allows us to better distinguish patients with a high and low degree of partial visibility of the glottis, which may provide a better assessment of the results obtained by the Miller and the Macintosh blades [20,21].

## 2. Materials and Methods

### 2.1. Study Settings

**Figure 1:**
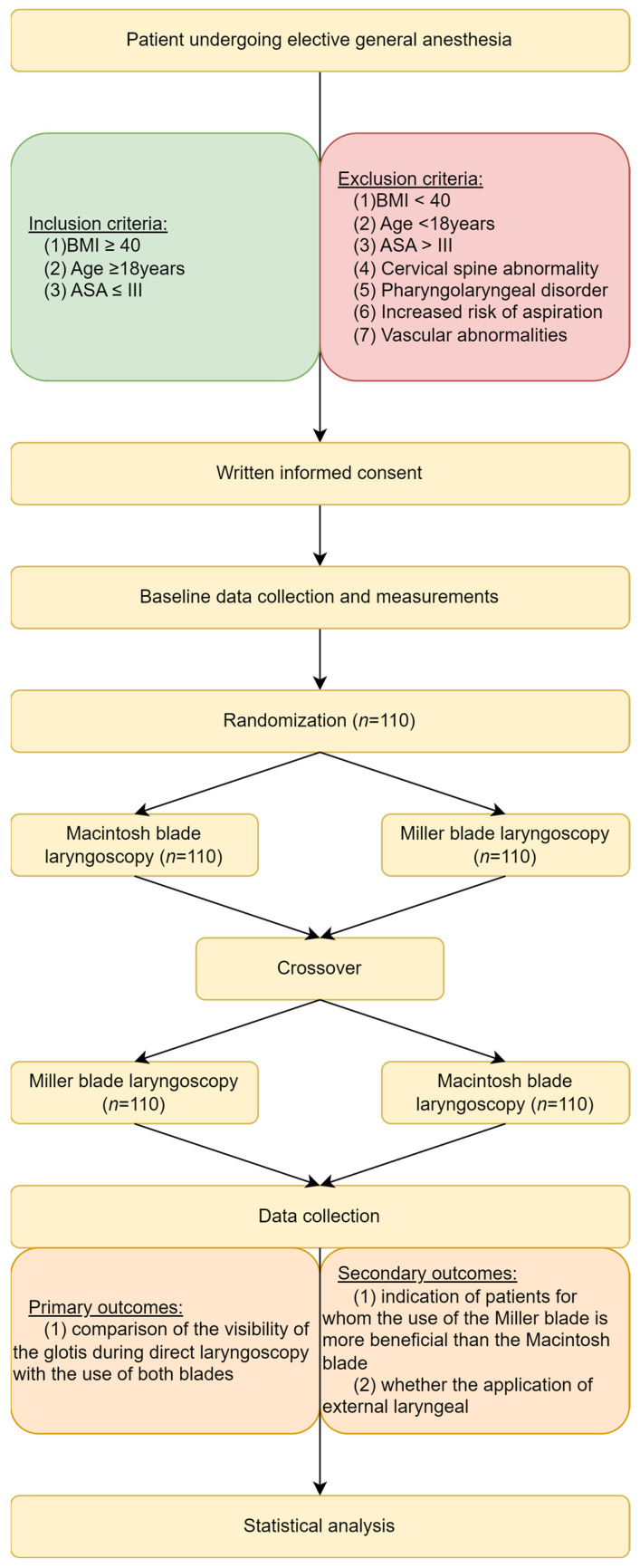
Flow chart of the present study.

The study was approved by the Bioethics Committee of the Medical University of Łódź (Number RNN/103/22/EC of 10.05.2022) and registered in Trial Registration (Number NCT05494463). It was carried out in the Central Operating Theatre of the Barlicki University Hospital No. 1 in Lodz, Poland. The direct laryngoscopy with an assessment of the glottis size and subsequent intubation was performed by anesthesiologists with over 20 years of experience intubating patients. Anesthesiologists participating in the study used Macintosh laryngoscopes more often in their daily professional practice and in the training process. This study is a randomized, controlled, blind trial with a parallel-crossover control. The order in which the blades were used was randomly selected using sealed opaque envelopes. The blind randomization strategy was generated using the Randomizer (randomizer.org) program (Figure 1).

The study enrolled 110 adult patients (over 18 years of age), with a BMI > 40, and with ASA ≤ III, planned for elective surgery under general anesthesia with endotracheal intubation. All patients gave informed and voluntary consent to participate in the study. Patients with: a BMI of <40, ASA IV and above, injuries of the cervical spine or scheduled for surgery on the cervical spine, an increased intraocular and/or intracranial pressure, vascular changes in the CNS and other parts of the body, diagnosed pathology of the respiratory tract after surgery of the oral cavity, pharynx or larynx, pregnant women, patients with indications of urgent surgical intervention or those not agreeing to participate in the study were excluded from participation in the study. In patients enrolled in the study, the following parameters were measured: body weight, height, BMI, neck circumference, thyromental distance, sternomental distance, mouth opening, and Mallampati scale. The Cormack–Lehane scale together with the percentage of glottis opening scale (POGO scale) were used to assess glottis visibility to allow a better assessment of airway entry in patients with partial glottis visibility. This may provide a better assessment of the results obtained by the Miller and Macintosh blade.

After transferring the first patient to the operating theater, the envelope was opened and the patient qualified for the first laryngoscopy using a random blade (Miller or Macintosh). Patients during the introduction of anesthesia were placed in a ramp-up position and monitored according to the standard: heart rate (HR), non-invasive pressure control (NIPC), and blood oxygen saturation (SpO_2_). Those qualified for the study were subjected to a unified technique of introduction to anesthesia: preoxygenation with 100% oxygen for 3 min, Fentanyl (FNT) 1.5 µg/kg iv., Propofol 2.5 mg/kg iv., and Rocuronium bromide 0.6 mg/kg iv., after adequate ventilation via face mask. Bag-mask ventilation with 100% oxygen and 2% inhaled sevoflurane was continued for 3 min. After obtaining a complete neuromuscular blockade, confirmed by the loss of a whole sequence of four responses using a peripheral nerve stimulator (Innervator Constant Current Peripheral Nerve Stimulator, Fisher & Paykel Health Care System, East Tāmaki, New Zealand), the intubating anesthesiologist performed the first direct laryngoscopy using a random Miller (Scope Medical Devices Pvt. Ltd., Ambala, India) or Macintosh blade (HEINE Optotechnik GmbH & Co. KG, Gilch-ing, Germany), and assessed the visibility of the glottis based on the CL scale and the percentage of glottis opening (PG scale) without external pressure on the larynx and with external compression. Afterward, another direct laryngoscopy was performed using the second blade with a following reassessment of glottis visibility based on both scales without pressure and with pressure in the same patient. Then, the patient was intubated and connected to an anesthesia machine. The obtained data have been saved.

Assessment of glottis visibility based on the CL scale and the PG scale without external compression and with compression on the larynx when performing direct laryngoscopy using the Miller and Macintosh blade was the primary endpoint of the study.

A secondary objective of the study was to find, among the measured parameters in patients with a BMI > 40, those parameters that may predict that the use of the Miller blade is associated with better visibility of the glottis gap than with the use of the Macintosh blade. A further objective was to assess whether the application of external pressure on the larynx improves the visualization of the glottis when using the Miller blade compared to the Macintosh blade in patients with a BMI > 40 kg/m^2^.

### 2.2. Safety Conditions

In addition to the experience of our team, we prepared all available devices for intubating patients with typical and so-called difficult airways. To prevent desaturation during direct laryngoscopy, we used high-flow O_2_ nasal cannula. This method is used to prolong the so-called safe apnea time during endotracheal intubation attempts. If any difficulties arise, the patient will be immediately intubated and connected to an anesthesia machine. In addition, devices for the so-called difficult airways were prepared, consisting of supralaryngeal instruments, video laryngoscopy, and endotracheal guides. To increase the patient’s safety in the event of unsuccessful intubation and to quickly reverse the neuromuscular blockade, Sugammdex has been prepared.

### 2.3. Statistical Analysis

We aimed to evaluate the visibility of the glottis during laryngoscopy with the Macintosh blade in the context of the following parameters used in common anesthesiological practice, namely: sex, age, weight, height, BMI, neck circumference, sternomental distance, thyromental distance, mouth opening, and assessment in the Mallampati score. As there is no single standard assessment of the visibility of the glottis, we used the following as the endpoints: better visualization of the CL scale (assessed with and without laryngeal pressure) and the PG scale (assessed with and without the laryngeal pressure).

Nominal data were presented as *n* (% of total) and assessed with a test chosen based on the size of the smallest subgroup: *n* < 5—Fisher exact test, 5 ≤ *n* < 15—Yates’s chi-squared test, and 15 < *n*—chi-squared test. Continuous data were tested with the Shapiro–Wilk test and Brown–Forsythe test. Data with normal distribution were presented as mean ± standard deviation and tested with parametric tests (Student’s *t*-test, Pearson correlation). In another case, they were presented as the median with interquartile range (IQR). Finally, we used receiver operating characteristic (ROC) analysis with Younden’s J-statistics to find an optimal cut-off point for qualification of intubation with Miller’s laryngoscope. The statistical analysis was performed using Statistica 13.1PL (StatSoft, Krakow, Poland).

## 3. Results

### 3.1. Study Group Characteristic

We collected data from 110 patients (65, 59.09% females) with the median age of 43 (IQR: 36–49.75) (Table 1).

### 3.2. Primary Endpoint

The use of the Miller blade resulted in a lower Cormac–Lehane score than the Macintosh blade: 45 (40.91%) without and 18 (16.36%) with laryngeal pressure. Similarly, higher POGO score was observed in 45 (40.91%) without and 19 (17.27%) with laryngeal pressure (Table 2A,B) Further, we assessed the parameters distinguishing patients with better a intubation result when compared to these with worse or the same results.

### 3.3. Secondary Endpoints

#### 3.3.1. Cormack–Lehane Scale without and with Laryngeal Pressure

The following parameters significantly differentiate patients with lower score in Cormack–Lehane scale when using the Miller blade vs. Macintosh blade, used without laryngeal pressure: neck circumference (46 (IQR: 42–49) cm vs. 42 (IQR: 41–46) cm; *p* = 0.016); weight (167 (IQR: 163–175) kg vs. 165 (IQR: 160–174) kg; *p* = 0.006); and BMI (47.33 (IQR: 44.43–50.94) vs. 44.98 (IQR: 42.76–48.33); *p* = 0.004). Similarly, the following factors were significantly different when laryngeal pressure was used: neck circumference (49.5 (IQR: 46.25–50.75) cm vs. 42 (IQR: 41–46) cm; *p* < 0.001); weight (150 (IQR: 137.75–157.75) kg vs. 127.5 (IQR: 116–138.25) kg; *p* < 0.001); height (173.5 (IQR: 167–175.75) kg vs. 165 (IQR: 161–173.25) kg; *p* = 0.037); and BMI (48.61 (IQR: 45.73–51.74) vs. 45.02 (IQR: 43.36–48.50); *p* = 0.021).

#### 3.3.2. POGO without and with Laryngeal Pressure

The following parameters significantly differentiate patients with a higher POGO score when using Miller blade vs. Macintosh blade without laryngeal pressure: neck circumference (46 (IQR: 42–49) vs. 42 (IQR: 41–46); *p* = 0.030); weight (135 (IQR: 124–148) vs. 126 (IQR: 115–139); *p* = 0.011); and BMI (47.33 (IQR: 44.43–50.81) vs. 44.98 (IQR: 42.76–48.56); *p* = 0.009). Similarly, the following factors were significantly different when laryngeal pressure were used: neck circumference (49 (IQR: 45–50.5) vs. 42 (IQR: 41–46); <0.001); weight (148 (IQR: 137–157.5) vs. 127 (IQR: 116–138.5); *p* < 0.001); and BMI (48.883604174924 (IQR: 45.75–51.77) vs. 44.99 (IQR: 43.36–48.47); *p* = 0.009).

#### 3.3.3. Optimal Cut-Off Points for Glottis Visualization When Using Miller Blade in Morbidly Obese Patients

**Figure 2:**
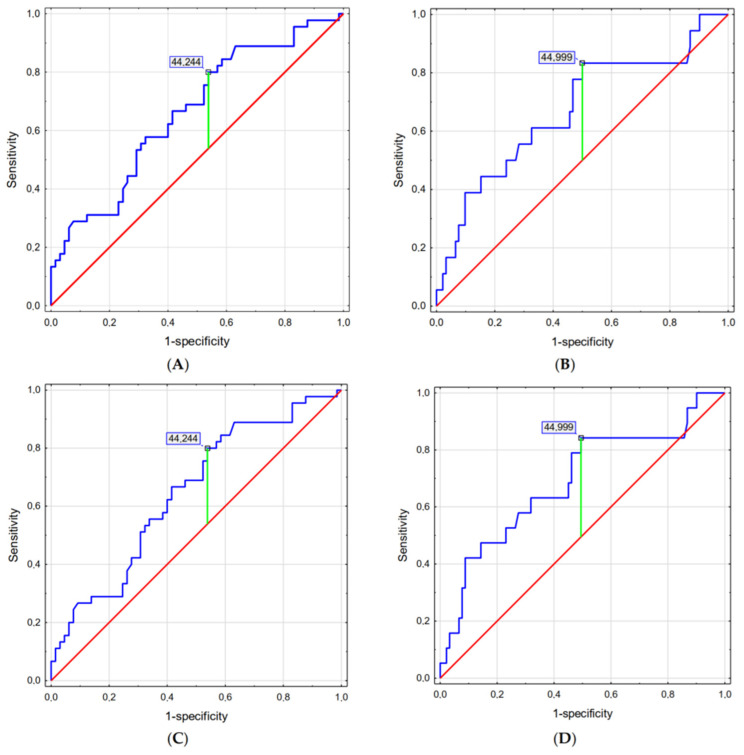
Optimal BMI cut-off point for intubation with Miller’s laryngoscope: (**A**) Cormack–Lehane scale without laryngeal pressure, (**B**) Cormack–Lehane scale with laryngeal pressure, (**C**) POGO without laryngeal pressure, (**D**) and POGO with laryngeal pressure. Legend: Blue line represents current model, red line represent curve for a random guess, green line represent the optimal cut-off point.

Miller’s laryngoscope was associated with a lower Cormack–Lehane score in obese patients and it should be considered especially in individuals with a BMI > 44.244 (without laryngeal pressure) and those with a BMI > 44.999 (with laryngeal pressure; Figure 2).

**Figure 3:**
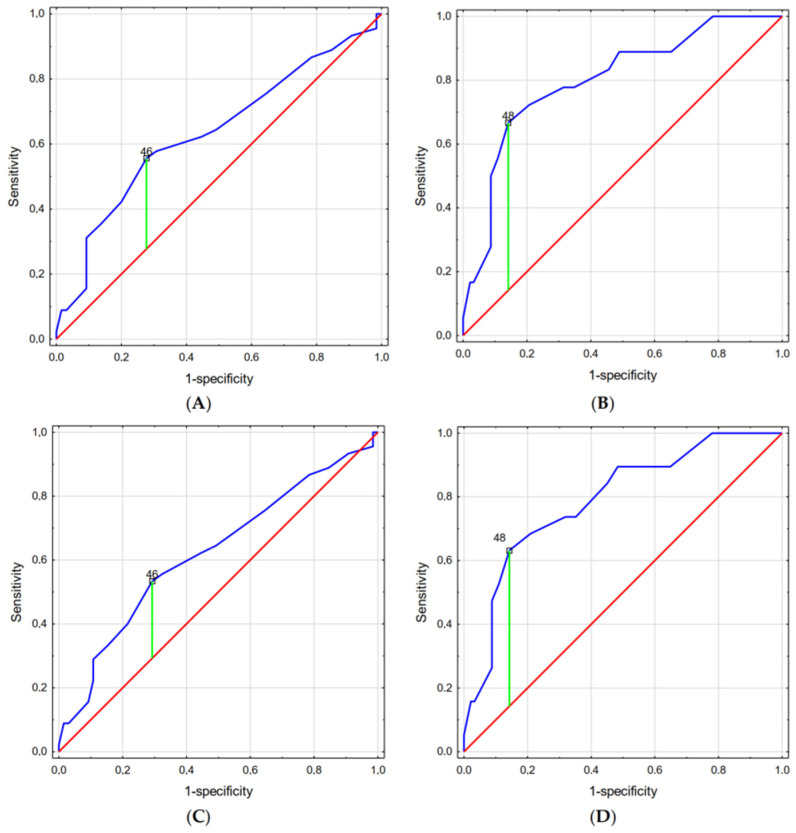
Optimal neck circumference cut-off points for intubation with Miller’s laryngoscope: (**A**) Cormack–Lehane scale without laryngeal pressure, (**B**) Cormack–Lehane scale with laryngeal pressure, (**C**) POGO without laryngeal pressure, and (**D**) POGO with laryngeal pressure. Legend: Blue line represents current model, red line represent curve for a random guess, green line represent the optimal cut-off point.

Similarly, Miller’s laryngoscope was associated with a higher POGO score for patients with a neck circumference higher than 46 cm (without laryngeal pressure) and 48 cm (with laryngeal pressure; Figure 3).

## 4. Discussion

The group of obese patients is a growing population often subjected to anesthesia in planned and acute settings [22]. According to the literature, difficult intubation can be expected more frequently in this group of patients than in the normal-weight population (14.3% vs. 3%; *p* = 003) [23]. The main factor determining the fast and safe opening of the airways is the correct visibility of the glottis. It is most often assessed using the Cormack–Lehane scale or the POGO scale. According to some authors, patients with increased body weight are prone to worse visibility of the airway opening, associated with a Cormack–Lehane score of 3–4 and lower on the POGO scale [2,19,20,21]. This is especially true for morbidly obese patients [1]. Many authors believe that the first choice in this group of patients should immediately be a video laryngoscope [2]. According to other authors, intubation should first be attempted with a Macintosh laryngoscope, which is as effective as a video laryngoscope when used by experienced incubators, and only in case of potential difficulties a video laryngoscope should be used. This could be due to the widespread use of the Macintosh laryngoscope and the still-limited use of video laryngoscopes, especially in poorer countries [24]. Some of the literature already states that the Miller laryngoscope provides better glottis visualization than the Macintosh laryngoscope [16]. It is often used for the intubation of pediatric patients, while its use in adult patients is limited [17]. According to Landry, who evaluated the use of Miller’s laryngoscope in a group of 978 adult patients, it is a highly rated device that provides good glottis visibility and effective intubation [18]. According to this author, the percentage of difficult laryngoscopy, scoring a 3–4 on the Cormack–Lehane scale associated with the use of Miller’s laryngoscope, is 6.8%. However, there is no data related to the use of the Miller blade in morbidly obese patients. Also, research on the visibility of the glottis gap that compares using both laryngoscopes in the same patients is yet to be conducted. In the available studies, the Cormack–Lehane scale or the POGO scale was most often used to assess the visibility of the glottis gap. In our study, we used both scales to compare the two blades and found that the Miller laryngoscope provided a higher percentage of morbidly obese patients with better visualization of the glottis, assessed using the Cormack–Lehane scale and the POGO scale (Table 2). This is consistent with Achen’s research, which showed that 25% of the visibility of the glottis gap was obtained in 95% of cases of laryngoscopy when using Miller’s laryngoscope. Using a Macintosh laryngoscope, only 25% of visibility was obtained when 80% of laryngoscopy was performed [25]. According to Arino, the Miller blade allows 96% visibility of the Cormack–Lehane epiglottal 1°, while in the case of the Macintosh laryngoscope, this percentage drops to 72% [26]. The better visibility, obtained with Miller’s laryngoscope, is associated with less movement of the cervical vertebrae and a smaller volume of tissue present in the mouth and throat that needs to be moved. When using a Macintosh blade to obtain the same view of the glottis as with a straight blade, the tongue must be shifted farther to the submandibular space. This is important in morbidly obese patients, in whom the accumulation of tissue in the oral cavity is greater than in patients with a normal body weight [16].

In our study, the use of external pressure on the larynx significantly improved the visualization of airway opening when using Miller’s laryngoscope in patients with greater BMI, neck circumference, body weight, and in the case of POGO scale, taller patients. According to the literature, external compression on the larynx improves glottis visibility and is necessary for 23% of Macintosh laryngoscopes and only 10% of Miller laryngoscopes [16,27]. Landry showed that problems regarding the view in ENT may occur in patients with a smaller disc–chin distance, higher scores on the modified Mallampati scale, and lack of prognostic capacity when using Miller’s laryngoscope [18].

According to Langeron et al., increased neck circumference and BMI > 35 kg/m^2^ are two independent indicators of potentially difficult intubation [3]. In such patients, minimizing intubation attempts is important to reducing the risk of complications [28]. In our study of patients with morbid obesity, the Miller blade provided a better view of the glottis as assessed together on the Cormack–Lehane scale and the POGO scale in patients with a BMI of >44.244 (or BMI > 44.999 when applying external compression to the larynx) and in those with a neck circumference of >46 cm (>48 cm using external compression). This is consistent with the conclusions reached by Kulkarni, who stated that the best laryngoscopic view is obtained with straight blades [16].

Conclusions: Usage of the Miller blade improves the visibility of the glottis compared to the Macintosh blade in morbidly obese patients. The recommendation to use a Miller’s blade in this group of patients requires further research, taking into account the effectiveness of intubation.

Limitations: Our research has several limitations. The most significant is the missing assessment of the effectiveness of Miller’s blade intubation in this group of subjects. Another is the relatively small group of examiners and the fact that the assessment of the visibility of the glottis gap was made only by very experienced anesthesiologists.

## Figures and Tables

**Table 1:** Study group characteristic encompassing parameters used in common anesthesiological practice.

Parameter	Value
Number of patients	110 (100%)
Sex [Female]	65 (59.09%)
Age [years]	43 (IQR: 36–49.75)
Neck circumference [cm]	44 (IQR: 41–47)
Sternomental distance [cm]	16 (IQR: 15–17)
Thyromental distance [cm]	6 (IQR: 6–8)
Mouth opening ≥ 4 cm:	71 (64.54%)
Mouth opening < 4 cm:	39 (35.46%)
Weight [kg]	130 (IQR: 118–145)
Height [cm]	166 (IQR: 162.25–175)
BMI [km/m^2^]	45.83 (IQR: 43.56–49.12)
Mallampati score	3 (IQR: 2–3)
Cormack–Lehane for Macintosh laryngoscope without laryngeal pressure	2 (IQR: 1–3)
Cormack–Lehane for Macintosh laryngoscope with laryngeal pressure	1 (IQR: 1–2)
Cormack–Lehane for Miller laryngoscope without laryngeal pressure	1 (IQR: 1–2)
Cormack–Lehane for Miller laryngoscope with laryngeal pressure	1 (IQR: 1–2)
POGO for Macintosh laryngoscope without laryngeal pressure	65% (IQR: 20–100%)
POGO for Macintosh laryngoscope with laryngeal pressure	100% (IQR: 50–100%)
POGO for Miller laryngoscope without laryngeal pressure	100% (IQR: 50–100%)
POGO for Miller laryngoscope with laryngeal pressure	100% (IQR: 50–100%)

**Table 2:** Intubation simplicity of Miller’s laryngoscope in comparison to Macintosh’s laryngoscope used without and with laryngeal pressure assessed using: (**A**) Cormack–Lehane and (**B**) POGO scales.

A. Cormack–Lehane Scale		
	Cormack–Lehane without Laryngeal Pressure	Cormack–Lehane with Laryngeal Pressure
Higher score	9 (8.18%)	12 (10.91%)
The same score	56 (50.91%)	80 (72.73%)
Lower score	45 (40.91%)	18 (16.36%)
**B. POGO Scale**		
	**POGO without Laryngeal Pressure**	**POGO with Laryngeal Pressure**
Lower score	13 (11.82%)	17 (15.45%)
The same score	52 (47.27%)	74 (67.27%)
Higher score	45 (40.91%)	19 (17.27%)

## Data Availability

Data available in authors depository.
